# Insight into genetic regulation of miRNA in mouse brain

**DOI:** 10.1186/s12864-019-6110-6

**Published:** 2019-11-13

**Authors:** Gordon Kordas, Pratyaydipta Rudra, Audrey Hendricks, Laura Saba, Katerina Kechris

**Affiliations:** 1 Department of Biostatistics and Informatics, Colorado School of Public Health, Aurora, CO 80045 USA; 2 Department of Statistics, Oklahoma State University, Stillwater, OK 74078-1056 USA; 3 Department of Mathematical and Statistical Sciences, University of Colorado Denver, Denver, CO 80217-3364 USA; 4 Department of Pharmaceutical Sciences, Skaggs School of Pharmacy and Pharmaceutical Sciences, University of Colorado Anschutz Medical Campus, Aurora, CO 80045 USA

**Keywords:** miRNA, eQTL, Hotspots, Mediation, Brain, Bayesian networks

## Abstract

**Background:**

micro RNA (miRNA) are important regulators of gene expression and may influence phenotypes and disease traits. The connection between genetics and miRNA expression can be determined through expression quantitative loci (eQTL) analysis, which has been extensively used in a variety of tissues, and in both human and model organisms. miRNA play an important role in brain-related diseases, but eQTL studies of miRNA in brain tissue are limited. We aim to catalog miRNA eQTL in brain tissue using miRNA expression measured on a recombinant inbred mouse panel. Because samples were collected without any intervention or treatment (naïve), the panel allows characterization of genetic influences on miRNAs’ expression levels.

We used brain RNA expression levels of 881 miRNA and 1416 genomic locations to identify miRNA eQTL. To address multiple testing, we employed permutation *p*-values and subsequent zero permutation p-value correction. We also investigated the underlying biology of miRNA regulation using additional analyses, including hotspot analysis to search for regions controlling multiple miRNAs, and Bayesian network analysis to identify scenarios where a miRNA mediates the association between genotype and mRNA expression. We used addiction related phenotypes to illustrate the utility of our results.

**Results:**

Thirty-eight miRNA eQTL were identified after appropriate multiple testing corrections. Ten of these miRNAs had target genes enriched for brain-related pathways and mapped to four miRNA eQTL hotspots. Bayesian network analysis revealed four biological networks relating genetic variation, miRNA expression and gene expression.

**Conclusions:**

Our extensive evaluation of miRNA eQTL provides valuable insight into the role of miRNA regulation in brain tissue. Our miRNA eQTL analysis and extended statistical exploration identifies miRNA candidates in brain for future study.

## Background

In recent years, there has been increasing interest in micro RNAs (miRNAs) [1]. miRNAs are small (approximately 22 nucleotides in length) non-coding RNA known to influence gene expression by way of targeting messenger RNA (mRNA). Specifically, miRNAs will act to repress mRNA translation or increase mRNA degradation [2]. miRNAs contain a small ‘seed’ region which is complementary to the 3′ untranslated region (UTR) of the mRNA(s) it targets [3]. More than 60% of human mRNA genes have such target sites in their 3′ UTR [4].

There are various miRNA biogenesis pathways [5]. The ‘canonical’ biogenesis of a miRNA starts with primary miRNA (pri-miRNA) being transcribed by either RNA polymerase II or RNA polymerase III. miRNA are transcribed from intronic regions (within a host gene) or from intergenic regions [6]. The pri-miRNA is further prepared by the Drosha microprocessor complex and the characteristic hairpin is cleaved by the Dicer complex [5]. The functional strand of the miRNA then combines with Argonaute proteins to form the RNA-induced silencing complex. This complex can then perform cleavage, promote translational repression, or deadenylate target mRNA [5]. At any point in this pathway there may be alterations or omissions that results in a non-linear pathway to a mature miRNA and thus, there exists various regulatory mechanisms of miRNA expression [5, 7]. miRNAs can be down-regulated or up-regulated and thereby, positively or negatively regulate gene expression respectively. miRNAs are important for cell development (including the vascular, immune, and neurological cells) [8]. miRNAs are also known to contribute to a wide variety of brain related diseases, including Alzheimer’s, Parkinson’s, Huntington’s and alcohol use disorders [8, 9].

The link between genetic background and miRNA expression can be investigated through expression quantitative trait loci (eQTL) analysis, which examines regions of the genome (loci) that influence a quantitative trait [10]. Here, the quantitative trait (i.e., continuous measure) is miRNA expression. Most frequently the regions of the genome are represented by single nucleotide polymorphisms (SNPs) [10]. eQTL can be placed in one of two categories depending on their genomic location. Local eQTL are located near the gene (or miRNA) while distal eQTL are in a region far from the gene (or miRNA). Local and distal are often referred to as *cis* or *trans*, where *cis* implies variants affecting transcription factor binding sites or other regulatory sequences near a gene, and *trans* implies variants affecting changes in the structure or function of transcription factors or other regulatory proteins for a more ‘global’ effect [11]. True *cis* effects are defined by Gilad as, “Regulatory elements [that] have an allele-specific effect on gene expression” [12]. Examples of *cis* regulatory elements include, promotors and enhancer elements [12]. We will assume that local implies *cis* and distal implies *trans*, but experimental validation is necessary to confirm these assumptions.

Many miRNA eQTL studies have been performed [13–19], but few examine miRNA specific to brain tissue [20, 21]. Cataloging brain tissue miRNA eQTL in mice provides a way to uncover genetic influence on miRNA expression levels that is difficult to determine in humans because of the challenges of obtaining brain tissue and difficulty in limiting the variability due to environmental exposure. Model organisms have the advantages of living in a controlled environment, and RNA samples from brain are easier to collect [22]. By combining the information from brain eQTL in mouse models, we can provide candidate miRNAs for future mechanistic studies in animals, which will serve as an accompaniment to the more limited human brain studies. Although in some cases specific mouse miRNA may not be conserved in humans, these miRNAs could still reveal biological mechanisms that are relevant in human. Furthermore, many miRNA eQTL studies have limited their scope to only *cis* eQTL [19, 21]. We will examine both *cis* and *trans* eQTL to gain more information on the regulation of miRNAs in brain.

The specific data used in this study are obtained from the LXS recombinant inbred (RI) panel. This panel was derived from the parental Inbred Long (L) Sleep and Inbred Short (S) Sleep strains [23], which were originally selected to vary in the loss of righting reflex (LORR) behavioral phenotype and were later inbred over many generations. The LORR phenotype is defined as the time it takes for a mouse to right itself in a v-shaped tray after being given a dose of ethanol [24]. Long sleep strains take a longer time to right themselves compared to the short sleep strains and are, therefore, more sensitive to the hypnotic effects of ethanol.

RI panels allow for improved mapping power due to their ability to minimize environmental variability and to isolate genetic variability by taking measurements on numerous mice from the same strain [23]. Another major advantage of the RI panel is that they are perpetually renewable and allow for the collection of many different traits by collaborating research teams over extended periods of time. The LXS panel is also useful for investigating variation in non-alcohol related traits, and has been shown to vary in phenotypes such as longevity [25], and hippocampus weight [26]. Furthermore, the advantage of using strains from a RI panel that have no experimental exposure (i.e., to ethanol) is that we can measure RNA expression levels that determine predisposition to a phenotype rather than expression levels that respond to an exposure.

We performed miRNA eQTL (mi-eQTL) analysis and mRNA, i.e. gene, eQTL (g-eQTL) analysis on the LXS RI panel to better understand the role of genetic regulation of miRNA expression in the brain. Related work included Rudra et al [24], which used the same miRNA brain expression data, but focused on a few specific alcohol related phenotypes, rather than taking a global approach. Therefore, our work is presented as a comprehensive QTL study that is generalizable to other brain related traits. This work helps fill the gap in mi-eQTL literature by providing resources specific to brain tissue, which is largely understudied. We also reported the results of a hotspot analysis, which has the potential to uncover novel regulators of miRNA expression. Finally, we integrated our results with available gene expression data on the same RI panel to examine the relationship between miRNAs and their associated gene targets via Bayesian network analysis. The extensive evaluation of mi-eQTL allows us to obtain more information on the role of miRNA regulation in brain and generate a resource for researchers investigating miRNA in brain and brain related diseases. Discovered mi-eQTL are available at PhenoGen (http://phenogen.org).

## Results

### mi-eQTL analysis

**Fig. 1:**
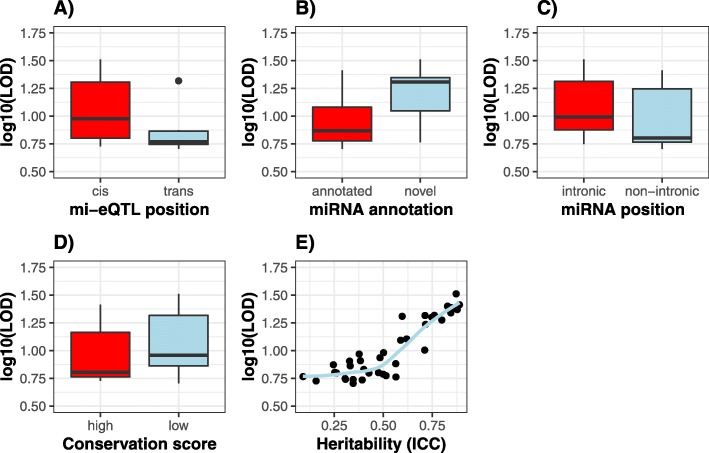
Comparisons of characteristics of mi-eQTL in brain with statistical significance. Log transformed LOD scores are for visualization reasons only. The actual calculations were done on untransformed LOD scores. **a** The difference in mi-eQTL strength between cis and trans mi-eQTL (Wilcoxon summed rank test-statistic (W) = 183, *p*-value = 0.023). **b** The difference in mi-eQTL strength between mi-eQTL of annotated miRNA compared to mi-eQTL of novel miRNA (W = 59, *p*-value = 0.028). **c** The difference in mi-eQTL strength between mi-eQTL with miRNA in intronic locations compared to those in non-intronic locations (W = 229, *p*-value = 0.067). **d** The difference in strength between mi-eQTL involving miRNAs that were highly conserved (mean PhastCon conservation score above 0.5) compared to those involving lowly conserved miRNAs (W = 108, p-value = 0.169). The conservation scores were dichotomized at 0.5 because that were often close to zero or one. **e** The relationship between mi-eQTL strength and the heritability (measured by the intraclass correlation coefficient) of the miRNA involved (in the mi-eQTL) (rho = 0.82, *p*-value = 3.67e-8)

mi-eQTL were obtained via correlation of miRNA expression and the genotype at a given genomic locus (see workflow in Additional file 1: Figure S3 and S4). Because of the multiplicity of SNPs across the RI panel, we test eQTL associations using strain distribution patterns (SDPs) (see Methods). Considering the power of our statistical tests due to the sample size and the nature of our permutation *p*-value calculation, each miRNA was limited to one genome-wide eQTL (across variants) represented by the maximum logarithm of the odds (LOD) score. The LOD Score is a representation of eQTL strength and allows us to compare different types of mi-eQTLs by their statistical strength (Fig. 1). 38 miRNAs (4.3% of all miRNAs tested) had a genome-wide significant mi-eQTL. Significance was determined via a permutation threshold of 0.05 to account for multiple testing across SDPs and further false discovery rate (FDR) threshold of 0.05 (to adjust for multiple testing across miRNAs). Table 1 contains all significant mi-eQTL and their corresponding Bayes’ 95% credible interval. All mi-eQTL tested can be found on PhenoGen (see Data Availability section) and Additional file 1: Figure S1 contains a visualization of eQTLs via a boxplot illustrating the differences in miRNA expression between genetic variant Eight (21%) miRNA involved in mi-eQTL were novel and 14 (37%) were miRNA transcribed from intronic regions (Table 2). The majority of mi-eQTL are *cis* mi-eQTL (79%), leaving only eight *trans* mi-eQTL (mmu-miR-677-5p, mmu-miR-193a-3p, mmu-miR-6929-3p, mmu-miR-6516-5p, mmu-miR-381-5p, mmu-miR-3086-5p, mmu-miR-32-3p, novel:chr4_10452). Human orthologs (of 8 miRNA) can be found in Additional file 1: Table S1.

**Table 1:** Significant brain mi-eQTL and their characteristics

miRNA	eQTL chr	eQTL location (Mb)	eQTL 95% C.I.	eQTL LOD	Genome-wide p-value	FDR	Cis/trans
mmu-miR-8114	1	154.3	(152.9, 154.9)	10.11	0.0010	0.0251	C
mmu-miR-32-3p	1	171.8	(171.1, 174.1)	5.51	0.0010	0.0251	T
mmu-miR-1981-3p	1	187.2	(183, 188.7)	7.63	0.0010	0.0251	C
mmu-miR-205-5p	1	193.6	(192.5, 193.6)	6.38	0.0010	0.0251	C
mmu-miR-669o-5p	2	9.8	(5.6, 10.9)	5.32	0.0020	0.0463	C
mmu-miR-467e-5p	2	10.6	(3.2, 10.9)	5.49	0.0010	0.0251	C
mmu-miR-669a-5p	2	10.6	(8.1, 10.9)	6.29	0.0010	0.0251	C
mmu-miR-297b-5p	2	10.6	(10.6, 10.9)	5.83	0.0020	0.0463	C
mmu-miR-7674-5p	2	32.8	(32.8, 34.6)	6.77	0.0010	0.0251	C
mmu-miR-466q	3	28.4	(28, 28.9)	18.86	0.0010	0.0251	C
novel:chr4_9669	4	43.1	(32, 43.1)	12.41	0.0010	0.0251	C
novel:chr4_11381	4	87.1	(87.1, 87.2)	23.47	0.0010	0.0251	C
mmu-miR-9769-3p	7	30.6	(30.6, 30.6)	25.92	0.0010	0.0251	C
mmu-miR-5121	7	43.5	(40.3, 45.5)	17.25	0.0010	0.0251	C
mmu-miR-7057-5p	7	64.6	(57, 67.1)	24.43	0.0010	0.0251	C
novel:chr8_23508	8	125.5	(125.4, 125.6)	21.89	0.0010	0.0251	C
novel:chr9_24385	9	77.3	(77.3, 77.3)	19.99	0.0010	0.0251	C
novel:chr4_10452	9	100.3	(94, 113.4)	5.78	0.0010	0.0251	T
novel:chr10_26214	10	4.8	(4, 5.6)	32.53	0.0010	0.0251	C
mmu-miR-6905-5p	10	25.3	(24.8, 25.9)	12.75	0.0010	0.0251	C
novel:chr10_26328	10	25.3	(24.8, 25.9)	20.74	0.0010	0.0251	C
mmu-miR-1934-5p	11	69.0	(67.8, 69.7)	20.39	0.0010	0.0251	C
mmu-miR-193a-3p	11	74.3	(74.3, 79.5)	7.29	0.0010	0.0251	T
mmu-miR-8103	11	95.1	(95.1, 99.6)	8.04	0.0010	0.0251	C
mmu-miR-152-5p	11	96.2	(93.4, 103.4)	6.33	0.0010	0.0251	C
mmu-miR-677-5p	11	98.3	(96.4, 101)	20.81	0.0010	0.0251	T
mmu-miR-5621-5p	11	115.6	(115.4, 116)	25.18	0.0010	0.0251	C
mmu-miR-208b-3p	14	54.7	(54.6, 55)	9.32	0.0010	0.0251	C
novel:chr15_40280	15	94.8	(94.8, 95.5)	8.10	0.0010	0.0251	C
mmu-miR-6516-5p	16	45.7	(45.7, 51.5)	5.94	0.0020	0.0463	T
mmu-miR-381-5p	16	45.8	(45.7, 51.5)	5.59	0.0010	0.0251	T
mmu-miR-6929-3p	19	30.0	(29.3, 30.5)	7.46	0.0010	0.0251	T
mmu-miR-3086-5p	19	30.2	(29.3, 30.5)	5.06	0.0010	0.0251	T
mmu-miR-201-5p	X	66.6	(65.4, 66.6)	8.63	0.0010	0.0251	C
mmu-miR-465c-5p	X	66.6	(65.4, 82.2)	5.44	0.0010	0.0251	C
mmu-miR-547-3p	X	66.6	(65.4, 66.6)	9.58	0.0010	0.0251	C
mmu-miR-871-3p	X	66.6	(49, 67.4)	6.28	0.0010	0.0251	C
mmu-miR-881-3p	X	66.6	(65.4, 67.4)	6.13	0.0010	0.0251	C

*Abbreviations: Chr* Chromosome*, pos* Position*, Mb* Megabase*, C.I.* Bayes’ credible interval*, LOD* Logarithm of the odds score, *FDR* False Discovery Rate, *cis/trans* cis (within 5 Mb on either side of the associated SDP) or trans (indicated by C or T)

**Table 2:** miRNA characteristics of those miRNA with significant mi-eQTL

miRNA	chr location	start location	end location	miRNA type	Anno- tation	conservation	ICC	No. targets
mmu-miR-8114	1	153899989	153900009	I	A	0.064	0.71	0
mmu-miR-32-3p	4	56895232	56895252	N	A	1.000	0.31	0
mmu-miR-1981-3p	1	184822409	184822429	I	A	0.062	0.56	0
mmu-miR-205-5p	1	193507503	193507524	N	A	1.000	0.25	31
mmu-miR-669o-5p	2	10514318	10514340	N	A	0.972	0.16	8
mmu-miR-467e-5p	2	10505731	10505752	N	A	NA	0.35	12
mmu-miR-669a-5p	2	10510185	10510208	N	A	NA	0.26	13
mmu-miR-297b-5p	2	10511686	10511707	N	A	0.727	0.09	147
mmu-miR-7674-5p	2	32050946	32050969	I	A	0.093	0.40	0
mmu-miR-466q	3	28419988	28420007	N	A	NA	0.80	175
novel:chr4_9669	4	41640264	41640319	N	N	0.525	0.59	0
novel:chr4_11381	4	87071780	87071841	I	N	0.264	0.88	0
mmu-miR-9769-3p	7	30552871	30552892	N	A	0.820	0.89	0
mmu-miR-5121	7	45126925	45126945	N	A	0.799	0.72	17
mmu-miR-7057-5p	7	66381702	66381719	I	A	0.000	0.85	0
novel:chr8_23508	8	125837774	125837841	N	N	0.003	0.84	0
novel:chr9_24385	9	74966743	74966804	I	N	0.474	0.75	0
novel:chr4_10452	4	132310004	132310065	N	N	0.822	0.56	0
novel:chr10_26214	10	4092814	4092873	I	N	0.002	0.87	0
mmu-miR-6905-5p	10	24910669	24910691	I	A	0.000	0.62	0
novel:chr10_26328	10	25416000	25416061	N	N	0.923	0.71	0
mmu-miR-1934-5p	11	69663055	69663077	N	A	0.000	0.60	13
mmu-miR-193a-3p	11	79712009	79712030	I	A	1.000	0.33	8
mmu-miR-8103	11	97063829	97063849	N	A	0.001	0.33	0
mmu-miR-152-5p	11	96850400	96850423	N	A	0.996	0.48	0
mmu-miR-677-5p	10	128085291	128085312	I	A	0.858	0.76	51
mmu-miR-5621-5p	11	115795824	115795846	N	A	0.003	0.83	0
mmu-miR-208b-3p	14	54975710	54975731	N	A	1.000	0.38	12
novel:chr15_40280	15	95488968	95489024	N	N	0.001	0.38	0
mmu-miR-6516-5p	11	117077370	117077391	N	A	0.974	0.51	0
mmu-miR-381-5p	12	109726829	109726851	I	A	1.000	0.31	0
mmu-miR-6929-3p	11	101419187	101419209	I	A	0.165	0.25	0
mmu-miR-3086-5p	19	58911725	58911744	N	A	0.012	0.35	9
mmu-miR-201-5p	X	67988135	67988156	I	A	0.001	0.48	31
mmu-miR-465c-5p	X	66832566	66832587	N	A	0.079	0.39	24
mmu-miR-547-3p	X	67988383	67988403	I	A	0.025	0.50	14
mmu-miR-871-3p	X	66810438	66810460	N	A	0.001	0.43	5
mmu-miR-881-3p	X	66801954	66801975	N	A	0.045	0.50	28

*Abbreviations: Chr* Chromosome*, annotation* Annotated or novel (indicated by A or N), where novel miRNAs were identified by the mirDeep2 software, *miRNA type* intronic or non-intronic (indicated by I or N) as determined by the UCSC Genome Table Browser, *conservation* PhastCons Conservation Score (closer to 1 indicates more highly conserved) where Not Applicable (NA) values indicate that a score was not returned by the Table Browser, *ICC* Intraclass correlation (a measure of miRNA heritability), *No. targets* Number of validated gene targets identified by the MultiMiR R package

*Cis* mi-eQTL compared to *trans* mi-eQTL have significantly higher LOD scores (p-value = 0.023; Fig. 1a). Additionally, novel miRNAs have significantly higher LOD scores on average, compared to annotated miRNAs (*p*-value = 0.028; Fig. 1b). However, there is no significant difference in mi-eQTL LOD score based on miRNA location (intronic versus non-intronic; Fig. 1c) or between highly conserved miRNAs and lowly conserved miRNAs (*p*-value = 0.169; Fig. 1d). The number of validated gene targets, as determined by MultiMiR [27] varied substantially between miRNAs (Table 2). Finally, we find a strong positive correlation between mi-eQTL LOD score and heritability of the miRNA involved (*p*-value = 3.67e-8; Fig. 1e).

### mi-eQTL enrichment analysis

We were only able to perform enrichment analysis on annotated miRNAs (30 of the 38 miRNAs with mi-eQTL). Of those 30 miRNAs, three had no related KEGG pathway information for their target genes, and 13 had less than four target genes with KEGG pathways information. Of the remaining 14 miRNAs with KEGG pathway information for at least four of their target genes, ten had brain-related KEGG pathways relevant to the nervous system, brain tissue, brain function or neurological/neuropsychiatric disease (Table 3). All results from the enrichment analysis can be found in Additional file 2.

**Table 3:** Brain-related enriched pathways obtained for annotated miRNA with a significant mi-eQTL

miRNA	Brain Related KEGG Pathway	# of genes	FDR
*miR-547-3p*	Axon guidance	5	0.0165
*mmu-miR-32-3p*	GABAergic synapse	7	< 0.0001
	Glutamatergic synapse	8	0.0007
	Nicotine addiction	5	0.0045
	Morphine addiction	6	0.0046
	Amphetamine addiction	7	0.0052
	Axon guidance	9	0.0095
*mmu-miR-208b-3p*	Glioma	5	0.0007
	Neurotrophin signaling pathway	6	0.0346
*mmu-miR-8114*	Axon guidance	5	0.0456
*mmu-miR-677-5p*	mTOR signaling pathway	13	0.0037
	Cocaine addiction	8	0.0273
*mmu-miR-6929-3p*	Ubiquitin mediated proteolysis	5	0.0208
*mmu-miR-465c-5p*	GABAergic synapse	9	< 0.0001
	Morphine addiction	10	< 0.0001
	Nicotine addiction	6	0.0028
*mmu-miR-193a-3p*	Glioma	4	0.0015
*mmu-miR-466q*	Nicotine addiction	4	0.0038
*mmu-miR-7674-5p*	Axon guidance	5	0.0010

FDR are the adjusted *p*-values. Only pathways with 4 or more genes and an FDR less than 5% are shown in the table. Pathways were deemed brain related if the PubMed search of the pathway name AND the keyword “brain” yielded at least one abstract. The abstract(s) were read to confirm brain related research

### Hotspot analysis

**Fig. 2:**
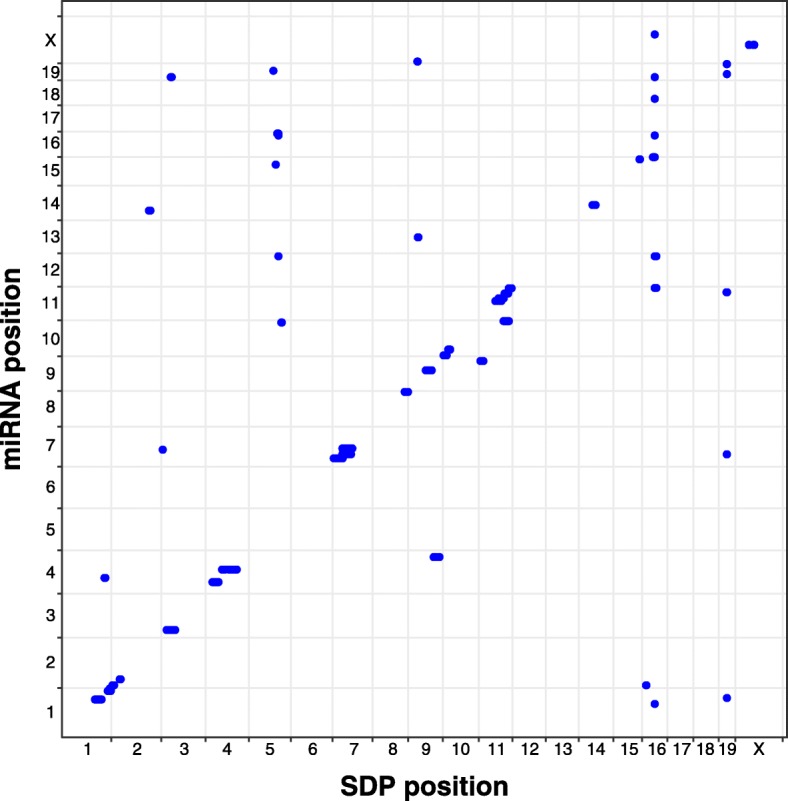
Chromosomal position of mi-eQTL. Rows are miRNAs and columns are SDPs. Scale is based on base pairs (bp). Blue spots indicate significant mi-eQTLs. A relaxed *p*-value threshold of 5e-6 is used to help illustrate potential hotspots

Figure 2 provides a visualization of the mi-eQTL analysis by physical location of the loci and of the miRNA. Although there are many cis mi-eQTL, indicated by points on the diagonal, there are also potential hotspots, indicated by vertical bands.

**Fig. 3:**
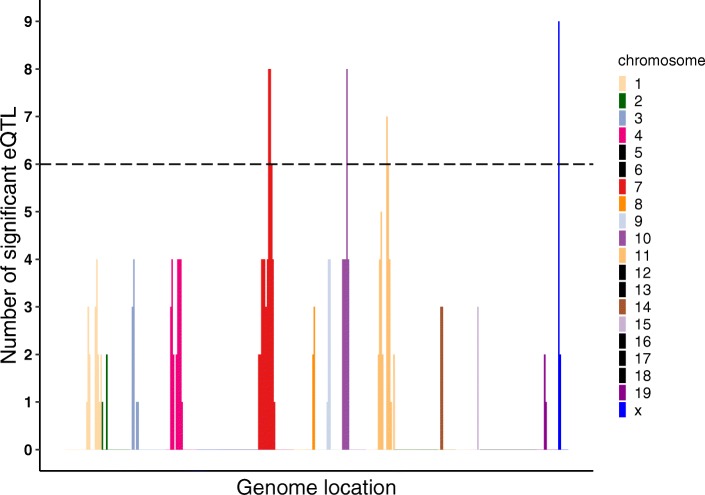
Brain mi-eQTL hotspots across the genome. Locations with more than 6 mi-eQTL cross the dotted line and indicate a significant hotspot. 6 is the threshold where the probability of getting more mi-eQTL in a bin is small (less than 0.05 after adjustments). Each color (as indicated by the legend) denotes the chromosome on which the significant mi-eQTL resides. Black in the legend denotes there were no significant mi-eQTL. The x-axis orders mi-eQTL from chromosome 1 up to chromosome X and is not scaled to physical distance

Potential hotspots were identified by dividing the genome into non-overlapping bins that were four SDPs wide (total number of bins equal to 354). Assuming mi-eQTLs were uniformly distributed across the genome, the counts of mi-eQTL in each bin follow a Poisson distribution [28]. To obtain a Bonferroni corrected *p*-value less than 0.05, a hotspot must have contained more than six mi-eQTLs. Using this cutoff, we identified seven bins with six or more mi-eQTL (see Fig. 3 and Table 4), that were collapsed into four final hotspots.

**Table 4:** Brain mi-eQTL hotspots and their respective locations

name	chromosome	start (Mb)	end (Mb)	number of mi-eQTL	Overlapping Addiction-related bQTL
Hotspot-chr7	7	37.8	64.6	22	Alcohol preference [29]
Hotspot-chr10	10	23.6	25.3	8	Morphine Preference [30]
Hotspot-chr11	11	93.4	102.6	13	LORR [31]
Hotspot-chrX	x	66.6	82.2	9	No Addiction Related

Hotspots were evaluated using mi-eQTL determined at a Bonferroni corrected threshold of 0.05 (4e-8) and hotspot significance of 0.05 after Bonferroni adjustment for number of bins tested. Overlapping bQTLs were determined by results of the MGI Phenotypes, Alleles, & Disease Models Search

There were originally two additional hotspots on chromosome 7 and one additional hotspot on chromosome 11 but they were collapsed with an adjacent hotspot (i.e. the ending SDP of the first hotspot resided directly next to the starting SDP of the second hotspot). Three of the four hotspots overlapped addiction related behavioral QTLs. We performed an enrichment analysis on the targets of any miRNA with mi-eQTL within a given hotspot using Diana-MirPath [32] (Additional file 1: Table S2). Of the nine miRNAs in the hotspots, seven had enrichment to a variety of functions including signaling and metabolism pathways.

### Bayesian network analysis

**Fig. 4:**
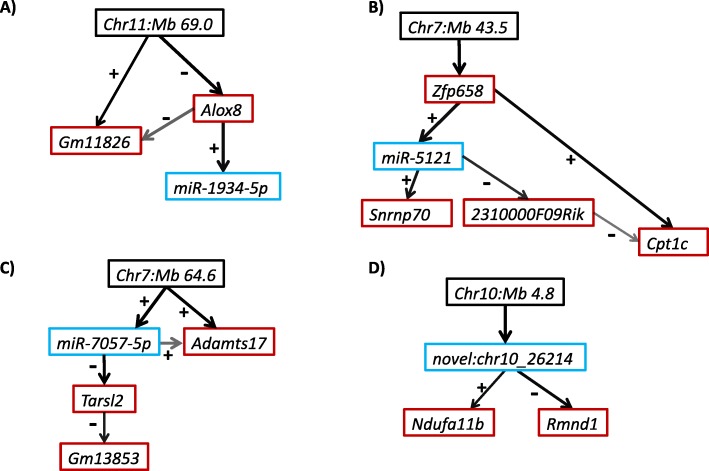
Bayesian networks of the four miRNAs. Using the hill-climbing algorithm, these were the networks determined by BIC and bootstrapping. A black box indicates the SDP location (associated with the miRNA), blue the miRNA, and red the genes. The thickness of the arrow shows the strength of association and the darkness of the arrow shows the strength of directionality as defined by the percent of the bootstrap iterations the edge or direction was observed, respectively. A plus sign next to an edge represents a positive association and a negative sign next to an edge represents a negative association (as determined by Spearman Correlation). **a** miR-1934-5p network **b** miR-5121 network **c** miR-7057-5p network **d** novel:chr10_26214 network

We tested triplets of SDP, miRNA, gene (i.e. mRNA) for evidence of mediation, where the association of the SDP with the miRNA (or gene) is mediated by a gene (or miRNA) respectively. Triplets were determined by the overlap of SDPs of the 38 significant mi-eQTL and SDPs of the 2389 significant g-eQTL (data not shown). Of the 175 possible triplets (SDPs, miRNA, mRNA), there were 11 significant triplets (*p* < 0.05) based on an initial mediation analysis (Additional file 1: Table S3). We then performed Bayesian Network Analysis (BNA) on these top mediation pathway candidates, which consist of four distinct miRNAs. Bayesian networks that included all genes and all miRNA associated with a given SDP were fit (Fig. 4).

The Bayesian network results identified two types of mediation for the four, candidate miRNAs. In one type of network, genes are acting as mediators of the effect of the genetic variant on miRNA expression (Fig. 4a, b), while in the other miRNAs are acting as mediators of the effect of the genetic variant on gene expression (Fig. 4c, d). The strength of associations was typically strong, as indicated by the thickness of the arrow (Fig. 4). In particular, 78% of all edges were contained in more than 80% of the bootstrap sample networks (Additional file 1: Table S4).

### Phenotypes

As an example of the utility of the mi-eQTL results, we evaluated the associations of mi-eQTL miRNAs with several alcohol related behavioral phenotypes including Sleep Time (ethanol and saline pre-treatment), Acute Functional Tolerance (ethanol and saline pre-treatment), and Rapid Tolerance from Bennett et al. [33]. Four miRNAs with a significant mi-eQTL had associations with phenotypes (FDR < 0.2), two with the Sleep Time and two with Acute Functional Tolerance (Table 5). The behavioral QTL (bQTL) for ST Saline on chromosome 4 overlaps with the mi-eQTL for novel:chr4_11381 (Table 5). In addition, the miRNA eQTL hotspots also overlapped with addiction-related bQTL (Table 4).

**Table 5:** miRNA associated with ethanol related phenotypes

miRNA	phenotype	Rho	FDR	bQTL chr	bQTL location (Mb)	bQTL *p*-value
novel:chr4_11381	ST Saline	−0.415	0.061	4	87.1	0.018
mmu-miR-32-3p	ST Saline	0.349	0.170	1	171.8	0.318
mmu-miR-208b-3p	AFT Ethanol	0.338	0.193	14	54.7	0.189
novel:chr4_10452	AFT Ethanol	0.343	0.193	9	100.3	0.206

Spearman correlation was used to determine associations. bQTL analysis was performed on the mi-eQTL locations of those miRNA associated with an ethanol related phenotype. ST Saline is the sleep time phenotype measured on mice pre-treated with saline. AFT Ethanol is the acute functional tolerance phenotype measured on mice pre-treated with ethanol. FDR is the false discovery rate. Only associations with FDR < 0.2 are shown

## Discussion

Protein coding gene expression has been the subject of most eQTL analyses, while mi-eQTL analyses have garnered less attention. These studies indicate that some eQTL are consistent across tissues, but other eQTL vary by tissue [34]. Because there are few eQTL analyses for miRNA, and because miRNA eQTL can vary by tissue [35], there is a need for tissue specific mi-eQTL studies. In particular, brain tissue has not been the subject of any genome-wide mi-eQTL analyses. In this work, we successfully identified and characterized significant mi-eQTL in brain tissue. We found hotspots and evidence of miRNAs as mediators of the genetic effects on gene expression. Furthermore, we established enrichment for brain related pathways among targets for miRNA with significant mi-eQTL. To our knowledge, this mi-eQTL study in mouse brain tissue is the most comprehensive genome-wide eQTL study to date.

Since miRNAs are regulators of steady state gene expression levels, the association between genetic differences and miRNA expression, as determined by mi-eQTL analysis, is relevant for identifying miRNAs that are important to gene regulation and may explain the genetic component of disease.

By examining features of the miRNA with mi-eQTL more closely, we may gain insight into the complex role that individual miRNA play in brain gene expression levels. In particular, we found that *cis* mi-eQTLs were significantly stronger than *trans* mi-eQTLs, which is consistent with cis eQTL generally being stronger than trans eQTL from g-eQTL analyses [36]. The significant correlation between mi-eQTL strength and miRNA heritability was also to be expected since large heritability indicates a strong overall genetic component for miRNA expression, and a strong mi-eQTL indicates a specific miRNA expression and genetic locus association [37]. Novel miRNAs were shown to have significantly stronger mi-eQTL as well.

Because there is limited knowledge about the factors that are important for tissue specific regulation of miRNA expression, we performed further analyses to gain deeper insight beyond just the discovery of individual mi-eQTL. Hotspot analysis is useful in identifying potential, ‘master regulators’ (one position in the genome that affects many miRNA) [38]. Many hotspot analyses have been performed on g-eQTL results [28, 39, 40] (see [38] for an entire list of gene hotspot studies), with fewer being performed on mi-eQTL results [13]. Identification of hotspots provides information on key loci that influence the expression of multiple miRNAs and subsequently the expression levels of genes targeted by those miRNAs. We discovered four hotspots in our analysis suggesting there are loci that control many miRNAs. These hotspots are especially important because miRNA expression hotspots in brain have not been well studied. Although the genes for Dicer and Drosha, which are important for the biogenesis of all miRNAs, were not physically contained by any of the hotspots, there may be other potential regulators for subsets of miRNAs.

To achieve an improved biological understanding of the mi-eQTL results, enrichment of miRNAs’ targets was performed. The targets of four of the miRNAs (*miR-547-3p, mmu-miR-32-3p, mmu-miR-8114,* and *mmu-miR-7674-5p*) with a significant mi-eQTL were individually enriched for the Axon Guidance KEGG pathway and the targets of four miRNAs (*mmu-miR-32-3p, mmu-miR-677-5p, mmu-miR-465c-5p,* and *mmu-miR-466q*) were enriched for addiction related pathways. Axon guidance is an integral part of the development of neural circuits. Improperly developed circuits can lead to Alzheimer’s or Parkinson’s disease [41]. Addiction pathways are also highly related to neuronal development in brain [42]. These enrichment results highlight the importance and specificity of miRNA in brain.

There were two miRNAs, miR-677-5p and miR-547-3p, that showed enrichment for brain related pathways and that were also involved in hotspots. miR-677-5p showed enrichment for the cocaine addiction and mTOR signaling pathways and was contained in Hotspot-chr11, which was also enriched for the mTOR signaling pathway. The mTOR pathway can be regulated by the drug Curcumin, and has been suggested as treatment for spinal cord injury (SCI) [43]. Additionally, Hotspot-chr11 overlaps with a bQTL for Loss of Righting Reflex (a phenotype that showcases the effects of ethanol) [31]. miR-547-3p was enriched for the axon guidance pathway, as previously discussed. miR-547-3p was associated with an SDP contained in Hotspot_chrX, which showed significant enrichment for morphine addiction, another brain specific pathway. The finding of these brain related functions suggests miRNA may influence predisposition to behavior or disease.

The connection between miRNA and mRNA expression is also important. To probe this connection, we combined multiple genes associated with a miRNA and a genetic variant in a directed network analysis. We identified two miRNA networks where the association between a genetic locus and gene expression is mediated by a miRNA, which suggests that the mediating effect of a miRNA is important to consider in gene eQTL studies. We also identified networks where genes may be mediating the association between a genetic locus and miRNA expression. The gene mediating networks may indicate indirect effects of genes regulating miRNAs.

Specifically, there were pathways mediated by miR-7057-5p and novel:chr10_26214 as shown in the Bayesian networks. miRNA novel:chr10_26214 is predicted to target genes Rmnd1 (required for meiotic nuclear division 1 homolog) and Ndufa11b (NADH:ubiquinone oxidoreductase subunit A11B) from chromosome 10 and miR-7057-5p mediates the relationship between chromosome 7 and Tarsl2 (threonyl-tRNA synthetase-like 2), which in turn Gm13853 (predicted gene 13,853) reacts to. miR-7057 has also appeared as a mediator of an alcohol related phenotype. There were also two pathways in which genes Alox8 (arachidonate 8-lipoxygenase) and Zfp658 (zinc finger protein 658) mediate the influence genetics on a miRNA.

Many of the genes involved in our Bayesian networks have a biological role in brain related diseases. Cpt1c (carnitine palmitoyltransferase 1c) is mainly expressed in neurons and has been shown to be associated with spastic paraplegia, a genetic disorder that causes leg stiffness and change in gait [44]. Snrnp70 (small nuclear ribonucleoprotein 70) encodes a protein that is associated with the formation of amyloid-beta plaques that contribute to the development of Alzheimer’s Disease [45]. Also, of importance, Tarsl2, partially encodes for aminoacyl-tRNA synthetases (ARSs) [46]. ARSs have been associated with several neuronal diseases [46].

As an example of the utility of our research, we investigated the connection between addiction related phenotypes and our results. We found four miRNA associated with the behavioral phenotypes we tested and an overlapping bQTL and mi-eQTL involving miRNA novel:chr4_11381 and the sleep time after pretreatment with saline (ST Saline) phenotype. Additionally, there were overlapping addiction related bQTL and hotspots, making those regions stronger candidates for further research.

There were a couple limitations to our study. First, as in most recombinant inbred panels, sample size is small and consequently, statistical power is limited. It is likely then, that weak (often the case for *trans* eQTL) mi-eQTL were not detected. However, the LXS panel is one of the largest mouse RI panels available. Second, both a potential drawback and advantage is the use of whole brain samples. On one hand, our results do not reflect a specific brain region, but as an advantage, they provide a general resource if the relevant brain region is not known. Finally, we were also unable to obtain enrichment pathways for novel miRNAs due to the lack of available annotation. Further investigations would need to be performed to confirm gene targets of the novel miRNAs.

The full mi-eQTL table can be found on PhenoGen (see Data Availability section). Researchers can use the mi-eQTL table to investigate a genomic location associated with a specific trait or disease and determine associated miRNA for that region. Alternatively, an investigator may start with a specific miRNA and check the mi-eQTL resource for evidence of a genetic association. These types of inquiries can identify candidate miRNAs and loci that are important for the regulation of a behavioral or disease phenotype and motivate future biochemical and mechanistic studies.

## Conclusions

Our results fill a deficiency in the mi-eQTL literature by providing resources specific to brain tissue. The hotspot analysis uncovered miRNAs that target biologically relevant genes in brain. Finally, by examining the relationship between miRNA expression and gene expression using Bayesian network analysis, we improve our understanding of how miRNAs may be associated with genetic variants and genes. This extensive evaluation of mi-eQTLs creates a platform for obtaining more information on the role of miRNA regulation in brain.

## Methods

### Animals

The LXS RI panel [47] was generated from crosses between the ILS and ISS strains of mice [24]. F2 mice pairs are then repeatedly inbred to create the inbred lines [24]. 175, group housed, male mice (59 LXS strains, 2–3 biological replicates per strain) were rapidly sacrificed using CO2 gas at approximately 10 weeks of age during the light phase, and brains were removed, divided sagittally, and placed in RNALater (Thermo Fisher Scientific) for RNA extraction and quantitation [24, 48]. All procedures for the Care and Use of Laboratory Animals were approved by the University of Colorado Boulder, IACUC. The procedures for RNA isolation were approved by the University of Colorado Anschutz Medical Campus IACUC.

### Genotype data

Genotype data on the LXS panel from Yang et al. [49] contains 34,642 informative SNPs excluding SNPs with missing data in at least one of the 59 strains used for analysis. Any number of SNPs can have the same SDP if they are in complete linkage disequilibrium [24]. If two SNPs have the same distribution of alleles across all strains, they have the same SDP. Since we only have 59 strains, many of the SNPs have the same pattern of variation. SNPs were compressed into SDPs to be computationally efficient. In total, we had 1416 SDPs, which were used for the mi-eQTL analysis. SDP locations are reported as the median SNP location of all SNPs that have an equivalent SDP.

### miRNA expression

miRNA expression data was obtained from animals bred at the Institute for Behavioral Genetics, Boulder, CO. RNA was obtained from whole brain tissue. Fragments in the 20–35 bp range were size selected to create the sequencing libraries. The Illumina HiSeq 2500 instrument was used to sequence single-end 50 base pair reads [24]. For mapping and quantification, we used a novel miRNA pipeline (miR-MaGiC) that allows for stringent mapping criteria because it maps to the individual transcriptome for each strain, and then further collapses miRNAs into, ‘miRNA families’ that allow for more accurate read quantification per miRNA (i.e., to avoid double read counting) [50]. The miRDeep2 software [51] was also implemented in order to identify novel miRNA by mapping reads to the genome. miRDeep2 first identifies an accumulation of reads that map to unannotated genome regions. Then, the region with reads and the regions that flank them are scored based on their probability to contain a secondary structure that resembles a miRNA precursor [51].

After mapping and quantitation, to remove batch effects and other unknown factors, we applied the Remove Unwanted Variations using residuals (RUVr) method [24, 52]. In total, 881 miRNAs remain, with 86 of them being novel [24]. To account for heteroskedasticity and dependence between the mean and variance, the Variance Stabilizing Transformation (VST) was used. The VST transformed expression data for individual mice was collapsed into strain averages [24]. We implemented VST via the DEseq2 (Version 1.22.2) package using the local dispersion fit parameter [53].

### Messenger RNA (mRNA) expression

Mouse whole brain mRNA expression data was obtained from the PhenoGen website [54], specifically as Affymetrix Mouse Exon 1.0 ST Array (Affymetrix, Santa Clara, CA) CEL files [24]. Probesets were filtered in accordance to the method of Vanderlinden et al. [55]. Probes that failed to align uniquely to the mouse genome or aligned to regions in the reference genome that contained a SNP for either of the parent strains compared to the reference genome were masked [55]. For probesets targeting the same gene, expression values were combined into a single expression value on the log base 2 scale using robust multi-array analysis (RMA) [24] within Affymetrix Power Tools [56]. Batch effects were adjusted for via the ComBat methodology [57]. mRNA samples were collapsed down to strain average means after keeping only the 59 strains that overlapped with the miRNA expression data.

### eQTL analysis

Following transformation of the count data via VST [58] and the calculation of strain means, expression quantitative trait loci analysis was performed using marker regression implemented using the R/qtl (Version 1.44.9) package [59]. In a marker regression analysis, expression is regressed onto the genotype. To be consistent with the literature [14, 16, 20] and the controlled nature of recombinant inbred mice (all of which are male), no covariates were included in the model. 95% Bayes’ credible intervals were also calculated using R/qtl. Credible intervals with zero width were expanded to the SDP’s widest SNP locations. Local eQTL are located within 5 Mb of the gene (or miRNA) while distal eQTL are in a region at least 5 Mb away from the gene (or miRNA) or on a separate chromosome [34]. We used the local and distal terminology interchangeably with *cis* and *trans* respectively.

We primarily focused on mi-eQTL, but g-eQTLs were also determined (see below). The complete workflow is presented in Additional file 1: Figure S3. Significant eQTLs were defined by permutation adjusted *p* values calculated in the R/qtl (Version 1.44.9) package [59]. One thousand permutations were used in the adjustment, and an alpha level of 0.05 was assumed. Due to limited power because of the sample size, mi-eQTL were limited to the eQTL with the maximum LOD score for each miRNA. Then, to correct for permutation *p*-values equal to 0, we implemented the Phipson and Smyth recommended estimate of exact p-values (adding one to both the numerator and denominator of the permutation p-value calculation) [60]. The permutation p-values account for the multiple testing across SDPs for each miRNA by permuting the strain labels. Note that this does not account for the multiple testing across miRNAs. Thus, multiple testing across miRNAs was controlled via a False Discovery Rate (FDR) threshold of 0.05 [61].

### miRNA with multiple locations

There are 32 miRNAs that have copies in multiple locations in the genome. To report a mi-eQTL, we must choose one location. Determining the best location for miRNA with multiple locations falls into three situations. In the most common situation, we decide based on the location with the strongest local eQTL (within 5 Mb on either side of the eQTL position [34]). If all possible locations fall into the same local window, then the location was chosen based on distance to the strongest SDP within the local window. Finally, if no SDPs fall within any of the local windows, then the location was chosen based on the shortest distance to the strongest SDP anywhere on the chromosome (Additional file 1: Figure S2).

### Evaluation of significant mi-eQTL

A variety of methods were used to evaluate significant mi-eQTL (see workflow in Additional file 1: Figure S4). Sequence conservation was determined using the PhastCon conservation score [62]. Scores for each miRNA involved in an eQTL were obtained from the UCSC genome browser Table browser tool using the Dec. 2011 (GRCm38/mm10) mouse reference genome and the 60 Vertebrate Conservation (Vert. Cons.) group of organisms for comparison. Scores were dichotomized using a cut-point of 0.5. Also, from the UCSC genome browser, both the same reference genome and Consensus Coding Sequences (CCDS) track were used to determine whether a miRNA was intronic. Heritability was estimated by calculating the intraclass correlation (ICC) using the HeritSeq (Version 1.0.1) package in R [37].

The multiMiR (Version 1.4.0) package [27] collates miRNA-target interactions derived from 11 external databases. From this software, we obtained both experimentally validated and computationally predicted miRNA gene targets. Predicted gene targets were only considered if the predictions were indicated by 3 or more databases.

### Enrichment analysis

Enriched pathways for both validated (Tarbase v7.0 [63]) and predicted (MicroT-CDS v5.0 [64]) gene targets of miRNA with eQTL were determined using the Diana-MiR Path bioinformatics tool [32]. KEGG Molecular pathways were investigated via the hypergeometric statistical test using an FDR correction for multiple testing [32]. Pathways were deemed brain related if the PubMed search of the pathway name AND the keyword “brain” yielded at least one abstract. The abstract(s) were read to confirm brain related research. Enrichment analysis on hotspots was performed on all miRNA targets associated with miRNA with mi-eQTL in a hotspot region.

### Hotspots

The two main approaches for hotspot detection are either permutations or based on bins [13, 28, 38, 39]. Since recombinant inbred strains have approximately a 50:50 allele frequency, permuting within SDPs is unnecessary. Therefore, we performed our hotspot analysis via the bin-based approach of Brem et al [28]. If the significant eQTL were uniformly distributed across the entire genome, then the number of eQTL within one bin (or window) would follow a Poisson distribution with mean and variance equal to the total number of eQTL divided by the total number of bins. Based on a Bonferroni corrected threshold of 0.05 (4e-8) on raw *p*-values and splitting the genome into 4 SDP wide bins, our Poisson mean was calculated to be 0.56. Using this threshold and Bonferroni correction for the number of bins, a hotspot must contain at least 6 eQTLs. Therefore, if the mi-eQTLs were randomly distributed across the entire genome then the probability of a bin containing more than 6 eQTLs is less than 0.05 adjusting for the number of bins tested. Sensitivity analysis with bin widths of 3 and 5 SDPs did not qualitatively change the results (data not shown).

### Bayesian network analysis (BNA)

We explored the relationships between genetic loci, and corresponding genes and miRNA in three steps. First, g-eQTL analysis was performed to determine associations between SDPs and genes (i.e. mRNA expression). Triplets of SDP, miRNA, gene (i.e. mRNA)) were initially identified by mi-eQTL and g-eQTL overlapping at a common SDP. Second, as a filter for Bayesian network analysis, we tested the triplets for evidence of (causal and reverse) mediation using the standard linear structural equation modeling (LSEM) method developed by Baron and Kenny was implemented [65].

Confidence intervals around the mediation coefficients were computed using the non-parametric bootstrap (1000 iterations) using the boot (Version 1.3.20) package [66, 67] in R. Due to the exploratory nature of the mediation analysis, 99.5% confidence intervals were determined, but no formal multiple testing correction was applied. Pathways were deemed significant if the confidence interval did not contain zero. Both miRNA expression and mRNA expression were evaluated as mediators.

Many significant triplets contained the same miRNA and different mRNA. Thus, for the third step, to estimate the direction of relationships among the many genes and the miRNA, Bayesian Networks [68] were fit using all genes implicated in a significant triplet with each miRNA. Gaussian Bayesian networks were fit using the hill-climbing algorithm [69] from the bnlearn (Version 4.4.1) package in R [70]. Network models were prioritized by the Bayesian Information Criteria (BIC). Edges were forced to be directed away from the SDP in all networks (since genetic variants are not influenced by either miRNA expression or mRNA expression). Edge strength was calculated by repeating the network learning process using 500 bootstrap samples of the original 59 strains. Network averaging was used to determine the final network structure (keeping a directed edge if observed in at least 50% of the bootstrap iterations) [70].

### Phenotypes

Associations between miRNA expression and LXS phenotypes were determined by Spearman correlation (corr.test in R) on strain means. As a use case we analyzed the Sleep Time with ethanol pre-treatment, Sleep Time with saline pre-treatment, Acute Functional Tolerance with ethanol pre-treatment, Acute Functional Tolerance with saline pre-treatment, and Rapid Tolerance phenotypes from the study conducted by Bennett et al. [33]. We performed bQTL analysis on the phenotypes associated with miRNA using the SDPs involved in their respective mi-eQTL. bQTL analysis was performed using simple linear regression in base R.

## Supplementary information

**Additional file 1.** Supplemental material that includes human orthologs, hotspot enrichment, mediation pathways, Bayesian network edge strength, an eQTL boxplot figure, two workflow figures, and miRNA location determination figure.

**Additional file 2.** KEGG enrichment pathways for all miRNA involved in significant mi-eQTL.

## Data Availability

Raw data on both miRNA expression and gene expression are available for download at https://phenogen.org/web/sysbio/resources.jsp?section=pub. miRNA expression data can also be found on the Gene Expression Omnibus (GEO) at https://www.ncbi.nlm.nih.gov/geo/query/acc.cgi?acc=GSE125953. The LXS exon array data can be found under the ‘Microarray’ tab and the LXS genotype data can be found under the ‘Genomic Marker’ tab. The full mi-eQTL table can be found at https://phenogen.org/web/sysbio/resources.jsp?section=pub&publication=210. The R-code to reproduce the analysis are available at https://github.com/gordonkordas/mirnabraineqtl.

## Abbreviations

BIC: Bayesian information criterion
BNA: Bayesian network analysis
bQTL: Behavioral quantitative trait loci
eQTL: Expression quantitative trait loci
g-eQTL: Gene expression quantitative trait loci
ILS: Inbred long sleep
ISS: Inbred short sleep
LOD: Logarithm of the odds
LORR: Loss of righting reflex
LS: Long sleep
mi-eQTL: MicroRNA expression quantitative loci
miRNA: MicroRNA
mRNA: Messenger RNA
RI: Recombinant inbred
SDP: Strain distribution pattern
SNP: Single nucleotide polymorphism
SS: Short sleep
UTR: Untranslated region
VST: Variance stabilizing transformation
