# Supplementary material for: Insight into genetic regulation of miRNA in mouse brain
Source: BMC Genomics. 2019 Nov 13;20:849. doi: 10.1186/s12864-019-6110-6 (PMC6854704; doi:10.1186/s12864-019-6110-6)
Supplement: Supplementary file 1 — Additional file 1. Supplemental material that includes human orthologs, hotspot enrichment, mediation pathways, Bayesian network edge strength, an eQTL boxplot figure, two workflow figures, and miRNA location determination figure. [file 12864_2019_6110_MOESM1_ESM.docx]

### Supplemental Tables and Figures for Insight into genetic regulation of miRNA in mouse brain

corresponding author: Gordon Kordas

**Tables**

**Table S1.** mi-eQTL miRNA with human orthologs.

| **Mouse miRNA** | **Human miRNA** |
| --- | --- |
| mmu-miR-152-5p | hsa-miR-152-5p |
| mmu-miR-193a-3p | hsa-miR-193a-3p |
| mmu-miR-205-5p | hsa-miR-205-3p |
| mmu-miR-208b-3p | hsa-miR-208b-3p |
| mmu-miR-297b-5p | hsa-miR-297 |
| mmu-miR-32-3p | hsa-miR-32-3p |
| mmu-miR-381-5p | hsa-miR-381-5p |
| mmu-miR-6516-5p | hsa-miR-6516-5p |

Human orthologs of miRNA involved in significant mi-eQTL as determined by miRbase (1).

**Table S2.** Hotspot enrichment.

| **Hotspot** | **miRNA** | **KEGG Pathway** | **# of genes** | **FDR**  **p-value** |
| --- | --- | --- | --- | --- |
| Hotspot-chr7 | *mmu-miR-5121* | NF-kappa B signaling pathway | 2 | 0.030544 |
|  | *mmu-miR-7057-5p* | Pancreatic secretion | 5 | 0.030544 |
|  |  |  |  |  |
| Hotspot-chr10 | *mmu-miR-6905-5p* | mRNA surveillance pathway | 4 | 0.009796 |
|  |  | ECM-receptor interaction | 1 | 0.009796 |
|  |  | Thyroid cancer | 1 | 0.031587 |
|  |  | Alcoholism | 3 | 0.040129 |
|  |  |  |  |  |
| Hotspot-chr11 | *mmu-miR-8103* | Hippo signaling pathway | 29 | 1.39E-06 |
|  | *mmu-miR-677-5p* | Lysine degradation | 9 | 0.000518 |
|  | *mmu-miR-152-5p* | Colorectal cancer | 13 | 0.000751 |
|  |  | Chronic myeloid leukemia | 16 | 0.001739 |
|  |  | Pathways in cancer | 46 | 0.004087 |
|  |  | Renal cell carcinoma | 12 | 0.005814 |
|  |  | Insulin signaling pathway | 24 | 0.006527 |
|  |  | Hepatitis B | 19 | 0.007321 |
|  |  | ECM-receptor interaction | 10 | 0.011257 |
|  |  | Arrhythmogenic right ventricular cardiomyopathy (ARVC) | 11 | 0.011257 |
|  |  | Tyrosine metabolism | 3 | 0.013062 |
|  |  | mTOR signaling pathway | 13 | 0.015629 |
|  |  | Other types of O-glycan biosynthesis | 6 | 0.015666 |
|  |  | Transcriptional misregulation in cancer | 25 | 0.015666 |
|  |  | Sulfur metabolism | 3 | 0.033553 |
|  |  | NF-kappa B signaling pathway | 10 | 0.033553 |
|  |  | HTLV-I infection | 33 | 0.033553 |
|  |  | Acute myeloid leukemia | 11 | 0.033869 |
|  |  | Ubiquitin mediated proteolysis | 22 | 0.039659 |
|  |  | Thyroid hormone signaling pathway | 16 | 0.039659 |
|  |  |  |  |  |
| Hotspot-chrX | *mmu-miR-547-3p* | Morphine addiction | 5 | 0.022326 |
|  | *mmu-miR-201-5p* |  |  |  |
|  | *mmu-miR-871-3p* |  |  |  |

FDR p-values are the FDR adjusted p-values at the 0.05 significance level. Pathways were enriched for non-novel miRNA. Specifically, novel-chr10_26328 was omitted from the Hotspot-chr10 enrichment.

**Table S3.** Mediation pathways.

| **miRNA** | **Gene** | **Chr** | **eQTL**  **loc. (Mb)** | **Mediation**  **Effect** | **99.5% C.I.** | **Prop. Med.** | **99.5% C.I.** |
| --- | --- | --- | --- | --- | --- | --- | --- |
| *miRNA as mediator* | | | | | | | |
| mmu-miR-1934-5p | Alox8 | 11 | 69.0 | -0.106 | (-0.172, -0.029) | 0.391 | (0.121, 0.737) |
| mmu-miR-1934-5p | Gm11826 | 11 | 69.0 | 0.254 | (0.042, 0.471) | 0.328 | (0.034, 0.7) |
| mmu-miR-5121 | Cpt1c | 7 | 43.5 | 0.038 | (0.004, 0.074) | 0.558 | (0.055, 0.97) |
| mmu-miR-5121 | Snrnp70 | 7 | 43.5 | 0.024 | (0.004, 0.053) | 0.721 | (0.197, 1) |
| mmu-miR-5121 | Zfp658 | 7 | 43.5 | 0.117 | (0.037, 0.163) | 0.480 | (0.119, 0.756) |
| mmu-miR-5121 | 2310002F09Rik | 7 | 43.5 | -0.081 | (-0.162, -0.005) | 0.598 | (0.089, 0.987) |
| mmu-miR-7057-5p | Adamts17 | 7 | 64.6 | 0.065 | (0.001, 0.115) | 0.552 | (0.041, 0.984) |
| mmu-miR-7057-5p | Gm13853 | 7 | 64.6 | 0.199 | (0.008, 0.421) | 0.850 | (0.103, 0.997) |
| novel:chr10_26214 | Ndufa11b | 10 | 4.8 | -0.267 | (-0.608, -0.034) | 0.778 | (0.169, 0.999) |
| novel:chr10_26214 | Rmnd1 | 10 | 4.8 | 0.088 | (0.001, 0.163) | 0.713 | (0.072, 0.999) |
| *Gene as mediator* | | | | | | | |
| mmu-miR-1934-5p | Gm11826 | 11 | 69.0 | -0.272 | (-0.508, -0.067) | 0.603 | (0.136, 0.989) |
| mmu-miR-1934-5p | Alox8 | 11 | 69.0 | -0.368 | (-0.561, -0.148) | 0.816 | (0.334, 0.999) |
| mmu-miR-5121 | Cpt1c | 7 | 43.5 | 0.237 | (0.035, 0.45) | 0.324 | (0.038, 0.632) |
| mmu-miR-5121 | Snrnp70 | 7 | 43.5 | 0.130 | (0.017, 0.321) | 0.178 | (0.039, 0.451) |
| mmu-miR-5121 | Zfp658 | 7 | 43.5 | 0.603 | (0.249, 0.806) | 0.826 | (0.298, 0.999) |
| mmu-miR-5121 | 2310002F09Rik | 7 | 43.5 | 0.164 | (0.006, 0.361) | 0.225 | (0.021, 0.559) |
| mmu-miR-7057-5p | Adamts17 | 7 | 64.6 | 0.285 | (0.003, 0.529) | 0.399 | (0.024, 0.728) |
| mmu-miR-7057-5p | Tarsl2 | 7 | 64.6 | 0.161 | (0.004, 0.303) | 0.226 | (0.016, 0.461) |
| mmu-miR-7057-5p | Gm13853 | 7 | 64.6 | 0.076 | (0.003, 0.175) | 0.107 | (0.007, 0.265) |
| novel:chr10_26214 | Rmnd1 | 10 | 4.8 | -0.176 | (-0.363, -0.001) | 0.199 | (0.016, 0.418) |
| novel:chr10_26214 | Ndufa11b | 10 | 4.8 | -0.082 | (-0.185, -0.007) | 0.092 | (0.006, 0.22) |

99.5% confidence intervals are bootstrap confidence intervals. Triplets were deemed significant if the confidence interval did not contain zero. Both mediation direction results are shown. *Abbreviations: Chr* chromosome, *Pos (Mb)* chromosomal position of SDP in megabases, *Prop. Med.* Proportion mediated.

**Table S4.** Bayesian network analysis edge strengths.

| **miRNA** | **Edge From** | **Edge To** | **Strength** | **Direction** |
| --- | --- | --- | --- | --- |
| *miR-1934-5p* |  |  |  |  |
|  | Alox8 | Gm11826 | 0.932 | 0.616 |
|  | Alox8 | miR-1934-5p | 0.956 | 0.951 |
|  | Chr11:Mb 69.0 | Alox8 | 1.000 | 1.000 |
|  | Chr11:Mb 69.0 | Gm11826 | 0.804 | 1.000 |
| *miR-5121* |  |  |  |  |
|  | Snrnp70 | Cpt1c | 0.874 | 0.974 |
|  | Snrnp70 | miR-5121 | 0.996 | 0.945 |
|  | 2310002F09Rik | Cpt1c | 0.682 | 0.553 |
|  | MiR-5121 | Zfp658 | 0.842 | 0.918 |
|  | miR-5121 | 2310002F09Rik | 0.782 | 0.836 |
|  | Chr7:Mb 43.5 | Snrnp70 | 1.000 | 1.000 |
| *miR-7057-5p* |  |  |  |  |
|  | Tarsl2 | Gm13853 | 0.774 | 0.889 |
|  | miR-7057-5p | Tarsl2 | 0.942 | 0.993 |
|  | miR-7057-5p | Adamts17 | 0.918 | 0.576 |
|  | Chr7:Mb 64.6 | Adamts17 | 0.888 | 1.000 |
|  | Chr7:Mb 64.6 | miR-7057-5p | 0.996 | 1.000 |
| *Novel:chr10_26214* |  |  |  |  |
|  | novel:chr10_26214 | Rmnd1 | 0.856 | 0.974 |
|  | novel:chr10_26214 | Ndufa11b | 0.748 | 0.944 |
|  | Chr10:Mb 4.8 | novel:chr10_26214 | 1.000 | 1.000 |

Edge strengths of the miRNA networks shown in Figure 4. Edge From indicates the origin of the edge and Edge To represents the destination of the edge. Edge strength represents the proportion of times the bootstrap samples contained the edge. Edge direction is the proportion of times the bootstrap samples contained the direction.

**Figures**

**Figure S1.** Boxplots illustrating the difference in miRNA expression for all miRNA with a mi-eQTL. Due to the differing expression levels of miRNA, the y-axis for each plot are not on the same scale. The peak SNP location labels the x-axis. Only two genotypes exist for recombinant inbred strains indicated by the parental allele. L represents the ILS strain and S represents the ISS strain. There are no heterozygotes in the LXS RI panel.

**Figure S2.** Location determination for miRNA with multiple locations. A) we decide final location based on the location with the strongest local (within 5 Mb on either side of the eQTL position) eQTL. B) If all possible locations fall into the same local window, then the location must be chosen based on distance to the strongest SDP within the local window. C) If no SDPs fall within any of the local windows, then the location must be chosen based on the shortest distance to the strongest SDP anywhere on the chromosome.

**Figure S3.** Mi-eQTL workflow. Blue boxes represent input data, gray boxes represent analytical steps and the red box represents the results. Full details of each step can be found in the eQTL analysis methods section.

**Figure S4.** Extended analysis workflow. Gray boxes represent analytical steps and the red box represents the results from the eQTL analyses. Full details of each step can be found in each of the analytical step’s respective methods section.

**References**

1. Kozomara A, Birgaoanu M, Griffiths-Jones S. miRBase: from microRNA sequences to function. Nucleic Acids Res. 2019;47(D1):D155-D62.
